# Development and Validation of a Predictive Model for Spontaneous Hemorrhagic Transformation After Ischemic Stroke

**DOI:** 10.3389/fneur.2021.747026

**Published:** 2021-11-15

**Authors:** Chenchen Wei, Junfeng Liu, Wen Guo, Yuxi Jin, Quhong Song, Yanan Wang, Chen Ye, Jing Li, Shanshan Zhang, Ming Liu

**Affiliations:** ^1^Department of Neurology, West China Hospital, Sichuan University, Chengdu, China; ^2^Department of Neurology, The Affiliated Hospital of Qingdao University, Qingdao, China; ^3^West China School of Medicine, Sichuan University, Chengdu, China; ^4^Department of Neurology, The First People's Hospital of Ziyang, Ziyang, China; ^5^Department of Neurology, Mianyang Central Hospital, Mianyang, China

**Keywords:** spontaneous hemorrhagic transformation, ischemic stroke, predictive model, atrial fibrillation, imaging factors

## Abstract

**Background:** Hemorrhagic transformation (HT) after reperfusion therapy for acute ischemic stroke (AIS) has been well studied; however, there is scarce research focusing on spontaneous HT (sHT). Spontaneous HT is no less important with a relatively high incidence and could be associated with neurological worsening. We aimed to develop and validate a simple and practical model to predict sHT after AIS (SHAIS) and compared the predictive value of the SHAIS score against the models of post-Reperfusion HT for sHT.

**Methods:** Patients with AIS admitted within 24 h of onset were prospectively screened to develop and validate the SHAIS score. The primary outcome was sHT during hospitalization (within 30 days after onset), and the secondary outcomes were symptomatic sHT and parenchymal hematoma (PH). Clinical information, laboratory, and neuroimaging data were screened to construct the SHAIS score. We selected six commonly used scales for predicting HT after reperfusion therapy and compared their predictive ability for sHT with the SHAIS score using Delong's test.

**Results:** The derivation cohort included 539 patients (mean age, 68.1 years; men, 61.4%), of whom 91 (16.9%) patients developed sHT with 25.3% (23/91) being symptomatic sHT and 62.6% (57/91) being PH. Five variables (atrial fibrillation, NIHSS score ≥ 10, hypodensity > 1/3 of middle cerebral artery territory, hyperdense artery sign, and anterior circulation infarction) composed the SHAIS score, which ranged from 0 to 11 points. The area under the receiver-operating characteristic curve (AUC) was 0.86 (95% CI 0.82–0.91, *p* < 0.001) for the overall sHT, 0.85 (95% CI 0.76–0.92, *p* < 0.001) for symptomatic sHT, and 0.89 (95% CI 0.85–0.94, *p* < 0.001) for PH. No evidence of miscalibration of the SHAIS score was found to predict the overall sHT (*p* = 0.19), symptomatic sHT (*p* = 0.44), and PH (*p* = 0.22). The internal (*n* = 245) and external validation cohorts (*n* = 200) depicted similar predictive performance compared to the derivation cohort. The SHAIS score had a higher AUC to predict sHT than any of the six pre-Existing models (*p* < 0.05).

**Conclusions:** The SHAIS score provides an easy-to-use model to predict sHT, which could help providers with decision-making about treatments with high bleeding risk, and to counsel patients and families on the baseline risk of HT, aligning expectations with probable outcomes.

## Introduction

Hemorrhagic transformation (HT) is a disastrous complication after acute ischemic stroke (AIS) with increased disability and mortality (1). The occurrence of HT is thought to be part of the natural evolution of AIS, and can be exacerbated by reperfusion therapy (2). It is widely recognized that the development of HT involves multiple and interconnected pathological processes (2, 3). The disruption of the blood-brain barrier is the essential pathogenesis of any HT, and the common molecular mechanisms underlying HT include the activation of reactive oxygen species and matrix metalloproteinases caused by cerebral ischemia or reperfusion injury, neuroinflammation, and vascular remodeling (4). There are some different mechanisms regarding the development of HT after reperfusion therapy. For example, the delayed reperfusion injury plays an important role in HT after endovascular treatment, and the direct toxicity of alteplase and alteplase-associated coagulopathy may accelerate HT after intravenous thrombolysis (3–5). Thus, it is of clinical significance to investigate the different types of HT separately due to their discrepancies in the pathophysiologic mechanisms.

There has been increasing interest in predicting HT recently. Some studies have reported several risk factors associated with HT after AIS (6, 7), and a few predictive models for the overall HT have been proposed (8, 9). In terms of the subtypes of HT based on the treatments, most recent studies have focused on HT after reperfusion treatments. However, <3% of AIS patients receive thrombolysis, and only 6% of patients were prescribed anticoagulants during hospitalization in China (10, 11). In other words, patients not treated with reperfusion therapy or anticoagulation treatment constitute the majority of AIS patients. In fact, predicting spontaneous HT (sHT) is no less important because the incidence of sHT reaches 12 to 43%, and up to 20% is associated with neurological worsening (12, 13). Therefore, research on sHT is also required urgently.

Some studies have investigated the risk factors for sHT (14–18). However, individual factors have limited predictive value. Several predictive models of HT after thrombolysis have been developed (19–23). Nevertheless, it is unclear whether these models are applicable for sHT, and there is scarce model especially proposed to predict sHT. Therefore, we aimed to (1) develop and validate a simple and practical score to predict sHT after AIS (SHAIS), and (2) compare the predictive ability of the SHAIS score against previous models of post-Reperfusion HT for sHT.

## Materials and Methods

### Patients

A consecutive cohort of patients with AIS admitted to the Department of Neurology, West China Hospital, Sichuan University, between January 1, 2016 and June 30, 2018, was prospectively screened as a derivation cohort, and patients admitted between January 1, 2014 and December 31, 2015 were retrospectively screened as an internal validation cohort. Patients with AIS who were admitted to Mianyang Central Hospital and The First People's Hospital of Ziyang between June 1, 2017 and March 31, 2019 were prospectively screened as an external validation cohort. Patients were eligible for the study if they (1) were at least 18 years old, (2) were admitted to hospital within 24 h of stroke onset, and (3) had an initial brain CT scan within 24 h after admission and follow-up CT or MRI during hospitalization. Patients were excluded if they received thrombolysis, endovascular treatment, or anticoagulation treatment during hospitalization for current stroke; had poor qualities of brain scans making the assessment of HT difficult; or presented with hemorrhage on the initial brain CT. Written informed consent was obtained from all the participants or their legal representatives. The project was approved by the Ethics Committee on Biomedical Research, West China Hospital of Sichuan University [2016 (339)], and the protocol followed local ethics criteria for human research.

The following data were collected: age, sex, prior medical history [hypertension, diabetes mellitus, hyperlipidemia, and atrial fibrillation (AF)], smoking, alcohol consumption, National Institutes of Health Stroke Scale (NIHSS) score, blood pressure and laboratory tests on admission (leukocyte count, platelet count, blood glucose, total cholesterol, triglyceride, high-density lipoprotein, and low-density lipoprotein), antiplatelet therapy, and the Trial of Org 10172 in Acute Stroke Treatment (TOAST) classification.

### Neuroimaging Scans

All patients underwent an initial CT scan within 24 h after admission, followed by a routine brain MRI during hospitalization (within 30 days after onset) or a repeated CT immediately whenever hemorrhage was suspected, such as in the case of headache or neurological deterioration. Three imaging factors were assessed on the initial CT scan, including the extent of hypodensity in the middle cerebral artery (MCA) territory, midline shift, and hyperdense artery sign. The extent of hypodensity was classified as whether the visible hypodensity involved >1/3 of the MCA territory. The midline shift was defined as midline shift of more than 5 mm at the septum pellucidum level or more than 2 mm at the pineal gland level (24). Hyperdense artery sign was defined as a vessel that appeared more hyper-attenuated than adjacent or equivalent contralateral arteries but non-Calcified (25). Two researchers independently read 30 randomly selected brain scans for the above imaging factors with an inter-rater kappa of 0.92 for hypodensity > 1/3 of the MCA territory, 0.70 for midline shift, and 0.86 for hyperdense artery sign. The corresponding intra-rater kappa was at least 0.88. One researcher assessed the remaining scans. We also divided AIS into anterior circulation infarction (ACI), posterior circulation infarction (PCI), or both ACI and PCI based on the infarct location.

### Definition of HT and Its Classifications

HT was defined as hemorrhage within the infarct territory or outside the infarct zone, which was detected on the follow-up brain scans, but not on baseline scan on admission (14). Based on the presence of associated neurological deterioration, HT was classified as symptomatic HT, when HT was accompanied by any decline in neurological status, or any clinical suspicion of hemorrhage was present; or as asymptomatic HT, when patients showed no worsening of neurological manifestations (26). According to the radiographic appearance of hemorrhage, HT was classified as hemorrhagic infarction, when hemorrhage presented as petechial infarction without space-occupying effect; or as parenchymal hematoma (PH), when hemorrhage (coagulum) occurred with mass effect (27).

### Construction and Validation of Predictive Model

We compared the differences in the baseline characteristics between groups of sHT and non-sHT in the derivation cohort, and then included variables with *p* < 0.05 in the univariate analysis into the stepwise backward logistic regression to identify the independent predictors of sHT. We chose to use stepwise backward logistic regression as this method theoretically simplifies the final model by eliminating variables with little predictive value (28). To develop a predictive model, we dichotomized continuous variables in the logistic regression. We assigned points for each of the variables based on the weight of its predictive value. The discriminatory ability and calibration of the newly proposed model for predicting sHT (i.e., the SHAIS score) were first assessed in the derivation cohort, and then were tested both in the internal and external validation cohorts. Since symptomatic HT and PH are of greater interest in clinical practice than asymptomatic HT and hemorrhagic infarction due to their close associations with poor outcomes (29, 30), we additionally tested the predictive value of the SHAIS score for symptomatic HT and PH as the secondary analysis.

### Selection of Predictive Models for HT After Reperfusion Treatment

We selected six predictive models of HT after thrombolysis or endovascular treatment: MSS (21), HAT (20), THRIVE (31), SEDAN (23), GRASPS (19), and SPAN-100 (22). All of them had been externally validated in different populations treated with thrombolysis (32–34). We excluded models such as SITS-ICH (35) because their components involved specific treatment, such as onset-to-alteplase time, which were unavailable for patients who did not receive thrombolysis.

### Statistical Analysis

Statistical analyses were performed using SPSS version 21.0, MedCalc version 15.2.2, and GraphPad Prism version 7.0. Results were presented as percentages, mean ± SD, median with inter-quartile range (IQR), or odds ratios (ORs) with 95% confidence intervals (CIs), as appropriate. Inter-group differences in continuous variables were assessed for significance using Student's *t* test or the Mann-Whitney U test; differences in categorical variables were assessed using χ^2^ or Fisher's exact test. The predictive ability (discriminatory ability) of the model was assessed using the area under the receiver-operating characteristic curve (AUC) with 95% CI. The 95% CI of AUC equals the AUC plus or minus 1.96 times the standard error of the area. The calibration of the model was evaluated with Hosmer-Lemeshow goodness-of-fit test. The maximal Youden index was used to determine the “optimal” cut-point of the model. Comparison of the predictive abilities (AUCs) between different predictive models was estimated using DeLong's test. A two-sided p < 0.05 was considered statistically significant.

## Results

### Patient Characteristics

From January 2016 to June 2018, 812 patients with ischemic stroke admitted to West China Hospital within 24 h of onset were screened, of whom 539 patients were included as a derivation cohort (Supplementary Figure 1). Mean age was 68.1 (±14.0) years, and 331 (61.4%) patients were men. The median hospital stay was 11 days (IQR 8–17 days), and the median time from stroke onset to follow-up imaging scans (i.e., the follow-up duration for sHT) was 4 days (IQR 3–8 days). For follow-up scans, 163 (30.2%) patients performed MRI but not CT, 71 (13.2%) patients only undertook CT, and 305 (56.6%) patients had both MRI and CT. During hospitalization, 91 (16.9%) patients developed sHT with 25.3% (23/91) being symptomatic sHT. The median time from stroke onset to detect sHT was 3 days (IQR 2–6 days), and the median time from onset to detect symptomatic sHT was 2 days (IQR 1–4 days). There were 49 patients with sHT diagnosed by MRI and 42 patients with sHT diagnosed by CT.

There were 245 patients included as an internal validation cohort, and 200 patients included as an external validation cohort (Supplementary Figure 1). In the internal validation cohort, the median follow-up duration for sHT was 7 days (IQR 4–9 days) after onset. A total of 37 (15.1%) patients developed sHT, of whom 7 patients suffered from symptomatic sHT. The median time from onset to detect sHT was 5 days (IQR 2–8 days), and the median time from onset to detect symptomatic sHT was 8 days (IQR 2–19 days). In the external validation cohort, the median follow-up duration for sHT was only 2 days (IQR 1–3 days) after onset. Spontaneous HT occurred in 15 (7.5%) patients, and the median time to detect sHT was 2 days (IQR 1–8 days). Only 4 patients developed symptomatic sHT, and all of them were detected within 3 days after stroke onset. Table 1 summarizes the characteristics of patients in each cohort.

**Table 1:** Characteristics of patients in the derivation and validation cohorts.

**Characteristics**	**Derivation cohort (*N* = 539)**	**Validation cohort**		*p*-value^*^
		**Internal**	**External**	
		**(*N* = 245)**	**(*N* = 200)**	
Male sex	331 (61.4)	135 (55.1)	130 (65.0)	0.09
Age, years	68.1 ± 14.0	66.8 ± 13.1	69.5 ± 11.8	0.12
**Comorbidities**				
Hypertension	293 (54.4)	163 (66.5)	121 (60.5)	0.005
Diabetes mellitus	119 (22.1)	65 (26.5)	36 (18.0)	0.10
Hyperlipidemia	17 (3.2)	40 (16.3)	13 (6.5)	<0.001
Atrial fibrillation	65 (12.1)	44 (18.0)	6 (3.0)	<0.001
Smokers	220 (40.8)	72 (29.4)	74 (37.0)	0.009
Drinkers	153 (28.4)	55 (22.4)	61 (30.5)	0.12
NIHSS score	6 (2–12)	4 (2–9)	4 (2–8)	<0.001
**TOAST classification**				<0.001
Artherosclerosis	149 (27.6)	56 (22.9)	117 (58.5)	
Small vessel occlusion	139 (25.8)	73 (29.8)	52 (26.0)	
Cardioembolic	114 (21.2)	26 (10.6)	13 (6.5)	
Other diseases	16 (3.0)	2 (0.8)	6 (3.0)	
Undetermined	121 (22.4)	88 (35.9)	12 (7.2)	
**Infarct location**				<0.001
ACI	445 (82.6)	167 (68.2)	135 (67.5)	
PCI	74 (13.7)	42 (17.7)	51 (25.5)	
Both ACI and PCI	20 (3.7)	36 (14.7)	14 (7.0)	
Antiplatelet therapy	513 (95.2)	227 (92.7)	198 (99.0)	0.007
**HT**	91 (16.9)	37 (15.1)	15 (7.5)	0.005
Symptomatic HT	23 (4.3)	7 (2.9)	4 (2.0)	0.27
PH	57 (10.6)	15 (6.1)	2 (1.0)	<0.001

*Data are shown as n (%), mean ± standard deviation, or median (interquartile range)*.

* *P-value was calculated using one-way ANOVA analysis or χ^2^ test. NIHSS, National Institutes of Health Stroke Scale; TOAST, Trial of Org 10172 in Acute Stroke Treatment; ACI, anterior circulation infarction; PCI, posterior circulation infarction; HT, hemorrhagic transformation; HI, hemorrhagic infarction; PH, parenchymal hematoma*.

### Development of the Predictive Model

The univariate analysis identified the following factors to be associated with sHT in the derivation cohort (Table 2): age, history of diabetes mellitus and AF, the baseline NIHSS score, systolic pressure on admission, TOAST classification, hypodensity > 1/3 of the MCA territory, midline shift, hyperdense artery sign, infarct location, platelet count, leukocyte count, total cholesterol, and low-density lipoprotein level. To construct the logistic regression model, we dichotomized the following continuous variables based on the nearest multiple-of-5 integer between the mean value or the median of each variable in HT vs. non-HT group: age (<70 years vs. ≥ 70 years), NIHSS score (<10 vs. ≥10), systolic pressure (<145 mmHg vs. ≥ 145 mmHg), platelet count (≤160 × 10^∧^9/L vs. >160 × 10^∧^9/L), leukocyte count (<8.00 × 10^∧^9/L vs. ≥ 8.00 × 10^∧^9/L), total cholesterol level (≤4.30 mmol/L vs. >4.30 mmol/L), and low-density lipoprotein level (≤2.50 mmol/L vs. > 2.50 mmol/L).

**Table 2:** Univariate analysis to identify potential predictors of spontaneous hemorrhagic transformation in the derivation cohort.

**Variables**	**HT**	**Non-HT**	***p-*value**
	**(*N* = 91)**	**(*N* = 448)**	
Male sex	51 (56.0)	280 (62.5)	0.25
Age, years	71.4 ± 12.8	67.4 ± 14.1	**0.01**
Hypertension	49 (53.8)	244 (54.5)	0.91
Diabetes mellitus	10 (11.0)	109 (24.3)	**0.005**
Hyperlipidemia	3 (3.3)	14 (3.1)	1.00
Atrial fibrillation	29 (31.9)	36 (8.0)	**<0.001**
NIHSS score on admission	14 (11–20)	4 (2–10)	**<0.001**
Systolic pressure on admission, mmHg	140.19 ± 21.49	149.40 ± 23.62	**0.001**
Diastolic pressure on admission, mmHg	82.67 ± 15.04	85.09 ± 15.32	0.17
**TOAST classification**			**<0.001**
Atherosclerosis	27 (29.7)	122 (27.2)	
Small vessel occlusion	0	139 (31.0)	
Cardioembolic stroke	44 (48.4)	70 (15.6)	
Other diseases	4 (4.4)	12 (2.7)	
Undetermined	16 (17.6)	105 (23.4)	
**Neuroimaging on initial CT**			
Hypodensity>1/3 of MCA territory	62 (68.1)	58 (12.9)	**<0.001**
Midline shift	3 (3.3)	1 (0.2)	**0.002**
Hyperdense artery sign	43 (47.3)	27 (6.0)	**<0.001**
**Infarct location**			**0.006**
ACI	79 (86.8)	366 (81.7)	
PCI	5 (5.5)	69 (15.4)	
Both ACI and PCI	7 (7.7)	13 (2.9)	
**Laboratory test**			
Platelet count, ×10^∧^9/L	152.65 ± 52.21	173.32 ± 65.75	**0.005**
Leukocyte count, ×10^∧^9/L	8.71 ± 3.07	7.87 ± 2.73	**0.009**
Glucose, mmol/L	8.37 ± 2.80	7.99 ± 3.34	0.32
Triglyceride, mmol/L	1.42 ± 1.08	1.67 ± 1.35	0.09
Total cholesterol, mmol/L	4.11 ± 0.91	4.45 ± 1.10	**0.003**
High-density lipoprotein, mmol/L	1.34 ± 0.42	1.28 ± 0.36	0.15
Low-density lipoprotein, mmol/L	2.38 ± 0.72	2.66 ± 0.91	**0.002**
Antiplatelet therapy	84 (92.3)	429 (95.8)	0.26

*Data are shown as n (%), mean ± standard deviation, or median (interquartile range). Boldface indicates statistical significance. HT, hemorrhagic transformation; NIHSS, National Institutes of Health Stroke Scale; TOAST, Trial of Org 10172 in Acute Stroke Treatment; ACI, anterior circulation infarction; PCI, posterior circulation infarction*.

**Figure 1:**
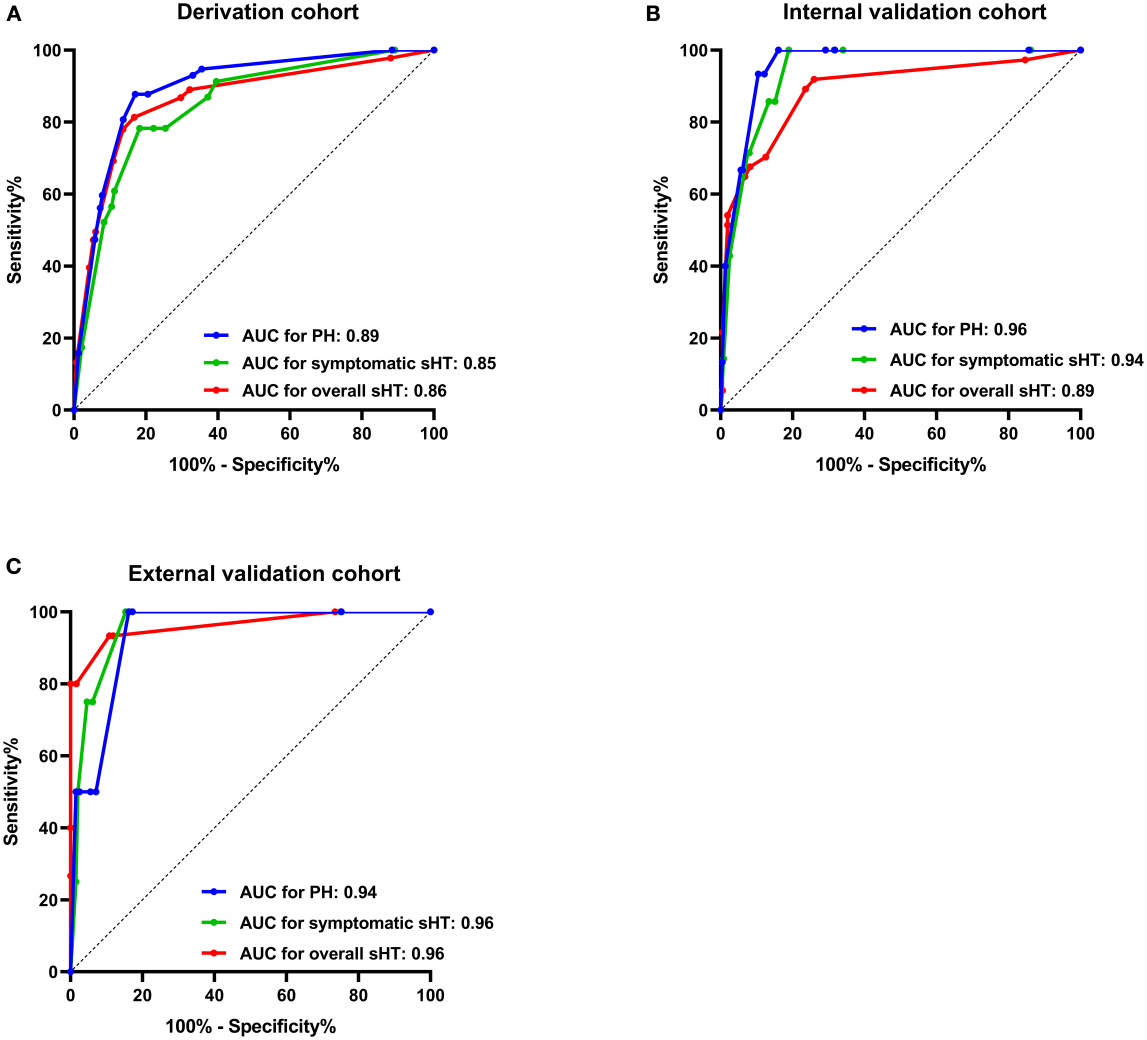
AUC of the SHAIS score for predicting sHT in the derivation cohort **(A)**, the internal validation cohort **(B)**, and the external validation cohort **(C)**. AUC, area under the receiver-operating characteristic curve; sHT, spontaneous hemorrhagic transformation; PH, parenchymal hematoma.

The stepwise backward logistic regression analysis identified the following independent predictors of sHT: AF, NIHSS score ≥ 10, hypodensity > 1/3 of the MCA territory, hyperdense artery sign, and infarct location (Table 3). Considering that the majority of sHT (86/91, 94.5%) occurred in ACI, the infarct location involving ACI was included as a component of the model. Finally, five components constituted the SHAIS score, which ranged from 0 to 11 points (Table 4, Supplementary Table 1). Logistic regression showed that every 1-point increase in SHAIS score was associated with an OR of 1.61 (95% CI 1.48–1.76, *p* < 0.001) for sHT. The AUC was 0.86 (95% CI 0.82–0.91, *p* < 0.001) for sHT (Figure 1). The SHAIS score >3 had the highest Youden index (0.65) to predict sHT among all the cut-off points. When we specified a score of 3 as the cut-off, the sensitivity and specificity were 81.3% and 83.3%, respectively. In terms of the calibration of the model, no evidence of lack of fit between the observed and predicted probabilities of sHT was found (*p* = 0.19). In terms of the subtypes of sHT, the AUC was 0.85 (95% CI 0.76–0.92, *p* < 0.001) for symptomatic sHT, and 0.89 (95% CI 0.85–0.94, *p* < 0.001) for PH (Figure 1). When the SHAIS score > 3, the sensitivity was 78.3% for symptomatic sHT and 87.7% for PH, while the specificity was 74.6 and 79.5%, respectively. No evidence of miscalibration of the SHAIS score was found to predict symptomatic sHT (*p* = 0.44) and PH (*p* = 0.22).

**Table 3:** Multivariate analysis to identify independent predictors of spontaneous hemorrhagic transformation in the derivation cohort.

**Variables**	**OR**	**95% CI**	***p-*value**
Atrial fibrillation	2.79	1.41–5.53	0.003
NIHSS ≥ 10	4.02	2.07–7.81	<0.001
Hypodensity > 1/3 of MCA territory	5.20	2.67–10.11	<0.001
Hyperdense artery sign	2.82	1.40–5.69	0.004
Infarct location	1.82	1.05–3.17	0.03

*Adjusted for age ≥ 70 years, diabetes mellitus, Trial of Org 10172 in Acute Stroke Treatment classification, systolic pressure ≥ 145 mmHg, midline shift, leukocyte count ≥ 8 × 10^∧^9/L, low-density lipoprotein ≤ 2.50 mmol/L, platelet count ≤ 160 × 10^∧^9/L, and total cholesterol ≤ 4.30 mmol/L. NIHSS, National Institutes of Health Stroke Scale; MCA, middle cerebral artery; OR, odds ratio; CI: confidence interval*.

**Table 4:** Score assignments of the predictive model of spontaneous hemorrhagic transformation.

**Components**	**Point**
**Atrial fibrillation**	
No	0
Yes	2
**NIHSS score on admission**	
≤ 9	0
≥10	2
**Hypodensity on initial head CT**	
≤ 1/3 of MCA territory	0
>1/3 of MCA territory	3
**Hyperdense artery sign on initial head CT**	
No	0
Yes	3
**Anterior circulation infarction**	
No	0
Yes	1
Total score	0–11

*CT, computed tomography; MCA, middle cerebral artery; NIHSS, National Institutes of Health Stroke Scale*.

### Validation of the Predictive Model

The AUCs of the SHAIS score in the internal validation cohort were 0.89 (95% CI 0.82–0.96) for the overall sHT, 0.94 (95% CI 0.89–0.99) for symptomatic sHT, and 0.96 (95% CI 0.93–0.99) for PH (Figure 1). The sensitivity and specificity of the SHAIS score > 3 to predict sHT were 70.3 and 87.5%, symptomatic sHT were 100 and 81.1%, and PH were 100 and 83.9%, respectively. No lack of fit between the score-expected rate and the observed rate of sHT was shown (overall sHT, *p* = 0.12; symptomatic sHT, *p* = 0.63; PH, *p* = 0.21).

In the external validation cohort, the AUCs for the overall sHT, symptomatic sHT, and PH were 0.96 (95% CI 0.90–1.00), 0.96 (95% CI 0.91–1.00), and 0.94 (95% CI 0.85–1.00), respectively (Figure 1). With a score of 3 as the cut-off, the sensitivity/specificity was 93.3/89.2% for the overall sHT, 100/84.7% for symptomatic sHT, and 100/83.8% for PH, respectively. Similarly, no lack of fit between the score-expected rate and the observed rate of sHT was found in the external validation cohort (overall sHT, *p* = 0.82; symptomatic sHT, *p* = 0.35; PH, *p* = 0.12).

### Comparison of Pre-existing Predictive Models

**Figure 2:**
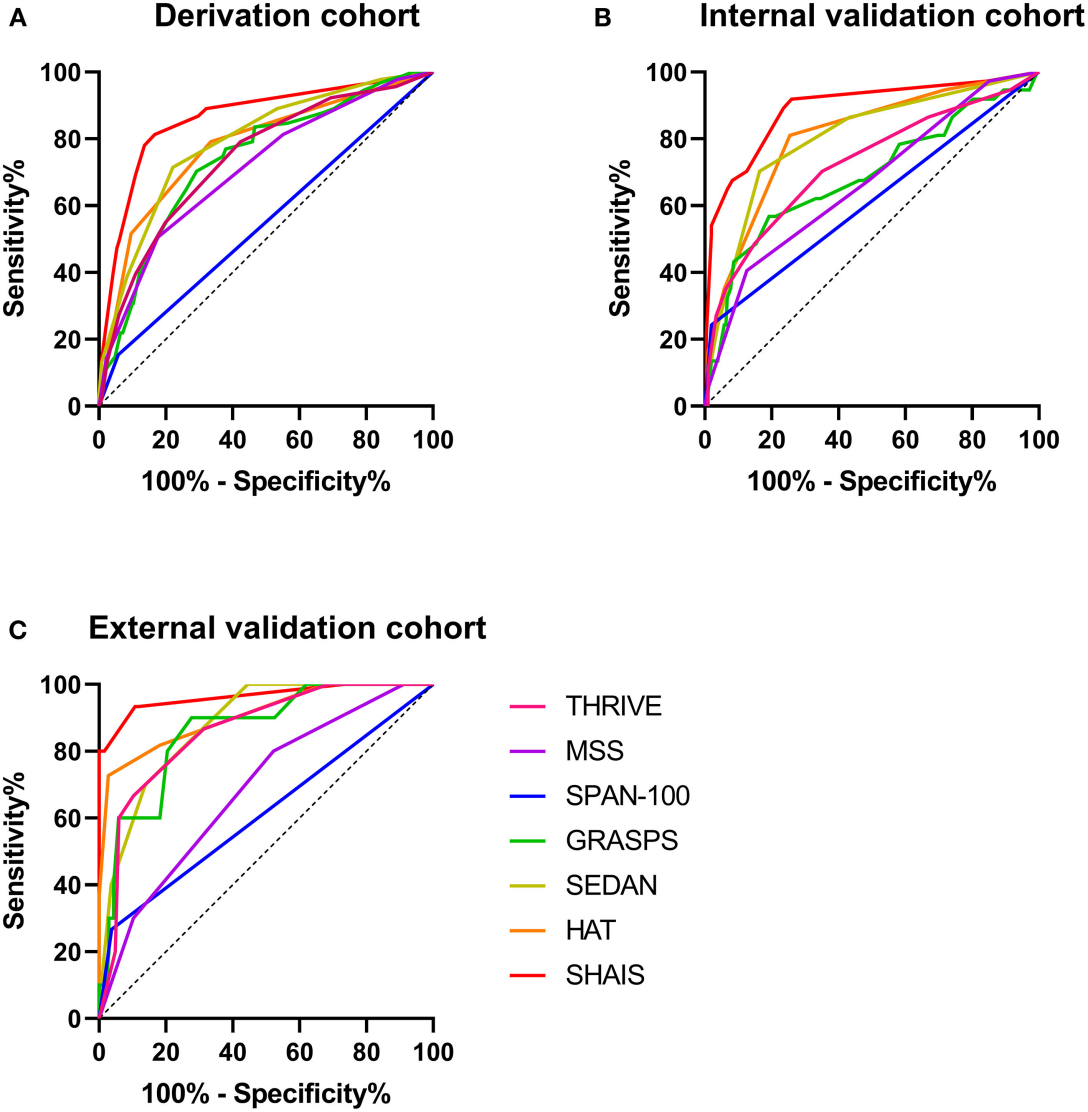
Comparison of AUCs of six previous models and SHAIS score for the overall sHT in the derivation cohort **(A)**, the internal validation cohort **(B)**, and the external validation cohort **(C)**. AUC, area under the receiver-operating characteristic curve; sHT, spontaneous hemorrhagic transformation.

Figure 2 illustrated the AUCs of the MSS, HAT, THRIVE, SEDAN, GRASPS, SPAN-100, and SHAIS score for the overall sHT in different cohorts. Since not all patients in the external validation cohort had the information of blood glucose and platelet count, several models involving blood glucose or platelet count (i.e., MSS, HAT, GRASPS, and SEDAN) could not be evaluated in patients with missing data. Therefore, the MSS score was available in 146 patients of the external validation cohort, the HAT score in 154 patients, the GRASPS score in 147 patients, and the SEDAN score in 147 patients.

Among the six previous models, the SEDAN score had the highest AUC of 0.79 (95% CI 0.74–0.84) for sHT in the derivation cohort. The HAT, MSS, THRIVE, and GRASPS scores showed similar predictive abilities for sHT (AUC 0.71–0.78). The AUC of SPAN-100 was only 0.55 (95% CI 0.48–0.62). The HAT score showed the best ability to predict the overall sHT both in the internal (AUC 0.81, 95% CI 0.73–0.89) and external (AUC 0.91, 95% CI 0.80-1.00) validation cohorts, while the SPAN-100 had the worst predictive ability (internal: AUC 0.61, 95% CI 0.50–0.72; external: AUC 0.61, 95% CI 0.45–0.78). The SHAIS score had a significantly higher AUC to predict sHT than any of the pre-Existing models in the derivation cohort and the internal validation cohort (all *p* < 0.05). The AUC of SHAIS was higher than the other scores in the external validation cohort, but there was no significant difference.

## Discussion

We developed an easy-to-use clinical score, the SHAIS score, with good predictive ability for sHT after AIS, and validated it internally and externally. Although the clinical characteristics of the derivation and validation cohorts were heterogeneous, the SHAIS score showed excellent predictive performance in each cohort, indicating that this score has a reliable and generalizable predictive capability. Furthermore, the ability of the SHAIS score to predict sHT outperformed other commonly used scores of HT after reperfusion therapy.

The ability to accurately predict sHT in the acute setting could help to counsel patients and families on the baseline risk of bleeding, aligning their expectations with probable outcomes. It could also help providers with decision-making about further treatments with high bleeding risk. Our study suggests that sHT is not uncommon after AIS even in the absence of reperfusion therapy and anticoagulation treatment, and that it can be symptomatic in approximately 3% of cases. We believe that sHT has its specific clinical significance and should be taken seriously. The SHAIS score utilized a selected few components that can accurately predict HT while maintaining computational simplicity. All these components are part of routine clinical assessment for patients with AIS upon admission, which can be easily and rapidly determined without expensive and advanced technologies, allowing the use of SHAIS score at small centers or community hospitals with limited resources.

The SHAIS score consisted of five factors: AF, NIHSS score, hyperdense artery sign, hypodensity of MCA territory, and ACI. Several researchers found that AF is a risk factor of sHT, which is in line with our results (14, 17). A study of 478 patients showed that every 1-point increase in NIHSS was associated with an OR of 1.05 for hemorrhagic infarction (6). The NIHSS score has been reported to be one of the factors most robustly associated with an increased risk of HT after reperfusion therapy (3). Our study revealed that NIHSS also played an important role in the development of sHT. The presence of hyperdense artery sign is thought to represent acute thrombus and is a surrogate of arterial obstruction (36). We did not evaluate the presence and extent of arterial stenosis or occlusion *per se* because angiography was not the primary examination upon admission for AIS patients, especially for those who were not eligible for reperfusion treatment. Several studies indicated that the presence of early CT signs and large infarct size were significantly related to the risk of sHT (14, 15, 18). Although both the 1/3 MCA rule and the Alberta Stroke Program Early CT Score (ASPECTS) on initial CT are widely used to assess the extent of early ischemic changes, the 1/3 MCA rule is easier to use for doctors in practice. Our data indicated that the predictive ability of the model including ASPECTS ≤ 6 was consistent with that of the model using hypodensity >1/3 of the MCA territory (data not shown). Therefore, we preferred including the 1/3 MCA rule as one component. The infarct location is another significant risk factor of sHT in our data set. The frequency of sHT in ACI was nearly three times higher than that in PCI (18.5 vs. 6.8%). Patients with ACI usually present with larger ischemic area and higher NIHSS score compared to those with PCI (37), and this may explain in part the association between infarct location and sHT.

In the current study, we validated the predictive value of previous models of HT after reperfusion therapy for sHT. This comparison was rational given that the development of sHT and post-Reperfusion HT involves some similar biological processes, such as ischemic injury, reperfusion injury, and the disruption of the blood-brain barrier, leading to some overlaps of the risk factors between the two types of HT (4). Our results showed that all these models had lower predictive ability for sHT than the SHAIS score, indicating that the accurate assessment of the risk of HT should use the specific predictive model based on whether the patients receive treatments with high bleeding risk. Some researchers found that post-Reperfusion HT has some different underlying pathological mechanisms from sHT (2–5), and these differences may partly explain why the predictive models of post-Reperfusion HT could not be generalized to the prediction of sHT. For instance, high reperfusion and collateral angiogenesis may be the main cause of HT after endovascular treatment (5). The toxicity of alteplase on the blood-brain barrier and the neurovascular units through effects on metalloproteinase activity and some receptor signaling is the particular pathogenic pathway of alteplase-related HT (4). Another determinant of alteplase-related HT is coagulopathy induced by alteplase itself, such as change in fibrinogen (3). In addition, most models of HT after thrombolysis included the elevated blood glucose level as one component (19–21, 23, 35). However, blood glucose and other laboratory tests such as leukocyte count and platelet count did not show a significant predictive power for sHT in our cohort. The role of hyperglycemia in sHT requires further investigation.

Our study has some limitations to be considered. The derivation and validation cohorts only consisted of Chinese patients. This may limit the generalizability of the results to non-Chinese populations. In addition, most patients with AIS in China would be administered Chinese medicines such as erigeron breviscapus to dilate blood vessels or reduce blood viscosity. Therefore, the prescription of Chinese medicines may increase the rate of sHT in our study and cause bias. Future studies are required to verify and extend the predictive value of the SHAIS score in other ethnicities. Symptomatic HT in our study was defined based on the National Institute of Neurological Disorders and Stroke criteria, but the recent guideline recommends assessing the degree of neurological worsening by NIHSS (3). However, we did not have the real-time NIHSS score when HT occurred, so we could not use other definitions of symptomatic HT such as the ECASS definition, which defines neurological deterioration as an increase of four points or more on the NIHSS (38). Although the SHAIS score was validated to show good predictive performance for both symptomatic sHT and PH in our study, this was a preliminary exploration based on a small number of patients with symptomatic HT and PH in the validation cohorts. Future research on these two specific subtypes of HT needs to enlarge the sample size to increase the statistical power of the analysis, and to choose a widely recognized and uniform definition of symptomatic HT to confirm the predictive ability of the SHAIS score. The detection of HT depended on baseline CT and follow-up MRI or CT during hospitalization. The difference between CT and MRI in the sensitivity to detect HT may result in potential bias. Moreover, the significance of MRI-detected HT still needs to be discussed in other studies. We encourage future work to choose a standardized imaging technology to diagnose HT. Besides, we used early hypodensity instead of exact infarct size as a component of the model because diffusion-weighted imaging (DWI) was not routinely and immediately performed in patients with AIS upon admission, and DWI lesion volume was unavailable in this analysis. It is notable that the goal of this study was not to define a new complex model but to identify a commonly used simple model for sHT. Future research can explore the weight value of exact infarct volume for the prediction of sHT if clinically feasible. Lastly, because CT is not accurate to evaluate ischemic change in infratentorial area, we only assessed early hypodensity in MCA territory. This may limit the use of the SHAIS score for extensive PCI. The accurate prediction for sHT in patients with PCI is required to be addressed in the future.

## Conclusions

We developed and validated an easy-to-use score to predict the risk of sHT during the first few days after AIS. Although the predictive model cannot be used as justification to withhold treatments from eligible patients, the SHAIS score may potentially help to guide providers and counsel patients and their families about the baseline risks of HT. Replication of our results and independent validation of the SHAIS score in a larger cohort is required before it can be applied to daily practice.

## Data Availability Statement

The data that support the findings of this study are available from the corresponding author on reasonable request.

## Ethics Statement

The studies involving human participants were reviewed and approved by the Ethics Committee on Biomedical Research, West China Hospital of Sichuan University. The patients/participants provided their written informed consent to participate in this study.

## Author Contributions

CW and ML conceived and designed the study. CW, JL, WG, YJ, QS, YW, CY, JL, and SZ collected the data and interpreted the neuroimaging. CW performed the statistical analysis and drafted the manuscript. JL contributed to critical revision of the manuscript. ML handled funding and supervision. All authors contributed to the article and approved the submitted version.

## Funding

This study was funded by the Major International (Regional) Joint Research Project, National Natural Science Foundation of China (Grant No. 81620108009) and National Natural Science Foundation of China (Grant No. 81901199).

## Conflict of Interest

The authors declare that the research was conducted in the absence of any commercial or financial relationships that could be construed as a potential conflict of interest.

## Publisher's Note

All claims expressed in this article are solely those of the authors and do not necessarily represent those of their affiliated organizations, or those of the publisher, the editors and the reviewers. Any product that may be evaluated in this article, or claim that may be made by its manufacturer, is not guaranteed or endorsed by the publisher.
