# Supplementary material for: Development and Validation of a Predictive Model for Spontaneous Hemorrhagic Transformation After Ischemic Stroke
Source: Front Neurol. 2021 Nov 15;12:747026. doi: 10.3389/fneur.2021.747026 (PMC8634397; doi:10.3389/fneur.2021.747026)
Supplement: Supplementary file 1 [file Data_Sheet_1.PDF]

SUPPLEMENTAL MATERIAL

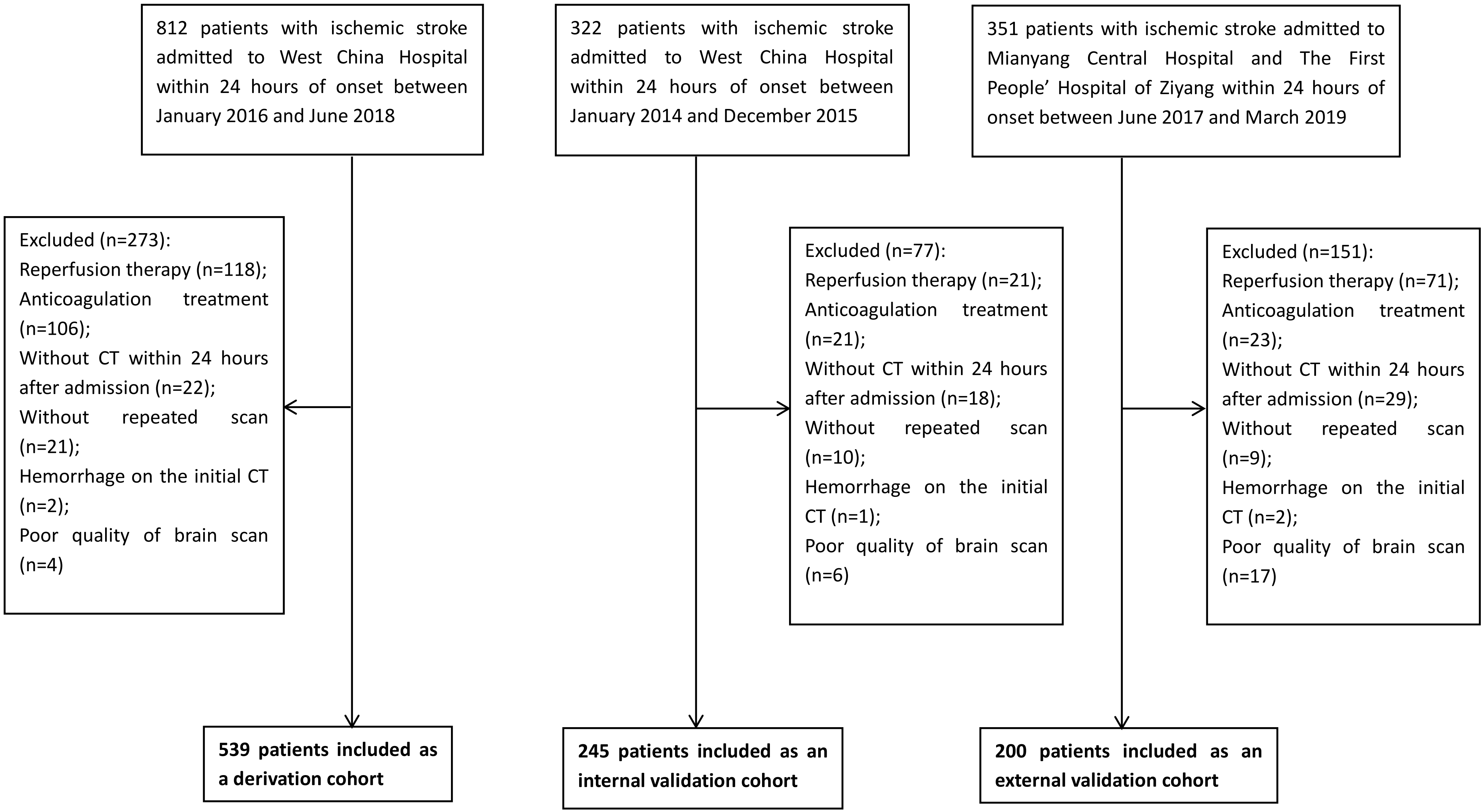

Supplemental Figure I Flowchart of patients inclusion

Supplemental Table I Distribution of the SHAIS score

The SHAIS Score	Number of Patients	Percentage of sHT
0	56	3.6% (2/56)
1	258	3.1% (8/258)
2	13	15.4% (2/13)
3	63	7.9% (5/63)
4	17	17.6% (3/17)
5	20	40.0% (8/20)
6	40	45.0% (18/40)
7	5	40.0% (2/5)
8	12	58.3% (7/12)
9	40	60.0% (24/40)
10	0	0
11	15	80.0% (12/15)
